# Comparison of Hemodynamic Parameters Based on the Administration of Remimazolam or Sevoflurane in Patients under General Anesthesia in the Beach Chair Position: A Single-Blinded Randomized Controlled Trial

**DOI:** 10.3390/jcm13082364

**Published:** 2024-04-18

**Authors:** Sangho Lee, Jimung Seo, Doh Yoon Kim, YoungYun Lee, Hee Yong Kang, Jeong-Hyun Choi, Youngsoon Kim, Mi Kyeong Kim, Ann Hee You

**Affiliations:** Department of Anesthesiology and Pain Medicine, Kyung Hee University College of Medicine, Kyung Hee University Hospital, Seoul 02447, Republic of Korea; sangholee@khu.ac.kr (S.L.); fobsop@khu.ac.kr (J.S.); 25494@khmc.or.kr (D.Y.K.); 26786@khmc.or.kr (Y.L.); hykang531@khu.ac.kr (H.Y.K.); cjh@khu.ac.kr (J.-H.C.); ys.kim@khu.ac.kr (Y.K.); mkanes@khu.ac.kr (M.K.K.)

**Keywords:** beach chair position, blood pressure, heart rate, hypotension, general anesthesia, pleth variability index, remimazolam, sevoflurane

## Abstract

**Background:** We aimed to evaluate whether the administration of remimazolam as a maintenance agent for general anesthesia affects the occurrence of hypotension compared with sevoflurane when switching to the beach chair position (BCP). **Methods:** We conducted a prospective randomized controlled trial from June 2023 to October 2023 in adult patients undergoing orthopedic surgery under general anesthesia in the BCP. A total of 78 participants were randomly allocated to the remimazolam (R) or sevoflurane (S) groups. The primary outcome was the incidence of hypotension that occurred immediately after switching to a BCP. The secondary outcomes included differences between the study groups in perioperative blood pressure (BP), heart rate (HR), endotracheal tube extubation time, postoperative complications, and hospital length of stay (LOS). **Results:** The incidence of hypotension immediately after switching to a BCP was significantly higher in the S group. The risk factors associated with hypotension included sevoflurane administration and a high baseline systolic BP. In the receiver operating characteristic curve analysis for the occurrence of hypotension after the transition to a BCP, the cutoff value for systolic BP was 142 mmHg. The perioperative BP and HR were higher in the R group at several timepoints. Postoperative endotracheal tube extubation time was shorter in the R group. There were no significant differences in the postoperative complications or hospital LOS between the two groups. **Conclusions:** Remimazolam should be considered as an anesthetic agent to prevent hypotension when switching to BCP, and hypotension may occur frequently in patients with high baseline BP.

## 1. Introduction

During clavicular, shoulder, or upper-arm surgery, the beach chair position (BCP) is preferred to access the surgical site and minimize damage to the upper arm plexus and blood vessels [1,2]. However, the transition from the supine to the BCP is accompanied by various hemodynamic changes. Owing to the influence of gravity, venous blood congestion occurs in the lower extremities; consequently, the preload, mean arterial pressure, stroke volume, cardiac output, and cerebral blood flow decrease [3]. These hemodynamic changes occur more prominently in patients under general anesthesia [4]. Hemodynamic deterioration may adversely affect postoperative outcomes, such as delayed neurological recovery [5,6]. Research has been conducted to minimize hemodynamic changes and improve the postoperative prognosis through fluid preloading or remote ischemic preconditioning [7,8].

Remimazolam is an ultrashort-acting benzodiazepine that is administered intravenously [9,10]. Similar to midazolam, it acts on γ-aminobutyric acid A receptors and is used to induce and maintain sedation and anesthesia [11]. Through a structural substitution similar to remifentanil, it is rapidly and independently metabolized by organs into CNS 7054, which is a pharmacologically inactive metabolite [12]. Its fast onset and offset, along with predictable duration, are advantageous. Similar to other benzodiazepines, the sedative effect can be reversed by flumazenil, and rapid awakening can be expected [13]. Several studies have confirmed the hemodynamic stability of remimazolam. Lee et al. retrospectively reported that in patients undergoing robotic gastrectomy, the remimazolam group required fewer vasopressors and showed hemodynamic stability compared to the sevoflurane group [14]. In a randomized study comparing propofol in patients who underwent endoscopic submucosal dissection, Qiu et al. reported that the remimazolam group had a lower incidence of hypotension, fewer vasopressor requirements, and a higher cardiac output [15]. Owing to these advantages, the use of remimazolam for anesthesia and sedation is increasing significantly.

The basic indicators of hemodynamic changes are blood pressure (BP) and heart rate (HR). Additionally, the pleth variability index (PVi; Masimo, Irvine, CA, USA), a relatively recently developed parameter, monitors continuous non-invasive fluid responsiveness. A PVi sensor was attached to the patient’s finger to monitor the relative variability in the photoplethysmograph and displays it as numerical values of 0–100 [16,17,18]. Existing fluid responsiveness parameters such as stroke volume variation (SVV) and pulse pressure variation (PPV) can only be monitored through invasive arterial cannulation; therefore, PVi allows for more convenient non-invasive monitoring.

However, to the best of our knowledge, no study has evaluated the hemodynamic stability using the PVi when switching from the supine position to the BCP with remimazolam. We hypothesized that the administration of remimazolam as a general anesthetic would reduce the incidence of hypotension during postural transition compared to the previously commonly used sevoflurane. We evaluated the effects of remimazolam on postoperative outcomes.

## 2. Materials and Methods

### 2.1. Study Design, Ethics, and Registry

This single-blind randomized controlled trial was designed to compare the incidence of hypotension based on the administration of remimazolam or sevoflurane in patients undergoing orthopedic surgery in the BCP under general anesthesia. Ethical approval was obtained from the Institutional Review Board of Kyung Hee University Hospital on 25 May 2023 (approval number: KHUH 2022-04-077) and written informed consent was obtained from all participants. This study was conducted in accordance with the principles of the Declaration of Helsinki. The trial was registered at the Clinical Research Information Service (KCT0008579, Principal investigator: Ann Hee You, Date of registration: 28 June 2023) prior to patient enrollment. The study protocol is available from Clinical Research Information Service. This study complied with the Consolidated Standards of Reporting Trials (CONSORT) checklist.

### 2.2. Participants

This study included patients aged 18–100 years who underwent orthopedic surgery in the BCP at a single tertiary medical center. The exclusion criteria were antihypertensive medications for hypertension, atrial fibrillation, body mass index (BMI) >35 kg/m^2^, pregnancy, American Society of Anesthesiologists physical status (ASA-PS) class ≥IV, and blood transfusion expected during surgery. Recruitment began in June 2023 and ended in October 2023.

### 2.3. Outcomes

The primary outcome was the incidence of hypotension immediately after switching to the BCP. Hypotension was defined as a decrease in systolic BP to <20% of the baseline BP, which was measured in the ward before arriving to the operating theater.

Secondary outcomes included perioperative differences in BP, HR, and PVi between the study groups. These hemodynamic parameters were measured at baseline, before tracheal intubation, before and after switching to BCP, 3 min after surgical incision, in the supine position after surgery, and in the post-anesthetic care unit (PACU). The total amount of intraoperative fluid and drug administered, estimated blood loss, and endotracheal tube extubation time were also measured. Extubation time was defined as the time from the administration of a neuromuscular blocker reversal agent to endotracheal tube extubation. Additionally, the time taken to recover to an Aldrete score of ≥9 in the PACU, postoperative nausea and vomiting (PONV), delirium, acute kidney injury (AKI), postoperative pulmonary complications (PPC) until postoperative day (POD) 2, and postoperative hospital length of stay (LOS) were recorded. AKI was defined as an elevation of serum creatinine level ≥0.3 mg/dL based on the Kidney Disease: Improving Global Outcomes criteria [19]. PPC was diagnosed based on changes in chest radiographic findings, respiratory infections, and oxygen demand [20].

Based on the medical records, the postoperative outcome assessor evaluated the results while masking group allocations and patient characteristics.

### 2.4. Procedures

Based on random allocation, the patients were divided into the remimazolam (R) and sevoflurane (S) groups. After entering the operating theater, a Masimo Radical 7 pulse oximeter probe (Masimo Radical 7; Masimo Corp., Irvine, CA, USA) was attached to the fourth finger of the non-surgical side to measure the PVi. BP was measured every 5 min using a non-invasive arm cuff on the same side. HR and rhythm were monitored using 3-lead electrocardiogram. The initial PVi, BP, HR, and peripheral oxygen saturation were recorded for all participants and preoxygenation was initiated. In the R group, 1 mg/mL remimazolam besylate (ByfavoTM, HANA PHARM CO. Ltd., Seoul, Korea) was prepared, and 0.1–0.2 mg/kg was administered intravenously for 1–2 min to achieve anesthesia induction. In the S group, a 1.5–2.0 mg/kg intravenous (IV) bolus of propofol was administered for anesthesia induction. In both groups, after loss of consciousness was confirmed, a neuromuscular blocker, rocuronium 0.8 mg/kg, was administered intravenously. Before tracheal intubation, a 0.5 µg/kg IV bolus of remifentanil was administered. For maintenance of anesthesia, 1–2 mg/kg/h of IV remimazolam was continuously infused in the R group, and sevoflurane was adjusted to maintain minimum alveolar concentration within 0.8–1.2 in the S group, based on the target range of the bispectral index (BIS), which was 30–60. In both groups, 0.03 mg/kg of IV midazolam was administered if the BIS level did not reach the target value. Before surgical incision, 0.3–0.5 µg/kg IV bolus of remifentanil was administered to reduce surgical pain. Intraoperatively, IV remifentanil was adjusted in a range of 0.05–0.2 µg/kg/min and to maintain within 20% of the initial BP and HR in both groups. If the BP and HR were unable to be maintained by remifentanil alone, beta-blockers (esmolol, 0.3 mg/kg, IV), vasodilators (nicardipine, 0.3–0.5 mg, IV), or vasopressors (ephedrine 5 mg or phenylephrine, 1 µg/kg, IV) were administered. The rate of intraoperative fluid administration through IV was set at 2–4 mL/kg/h. Remifentanil, remimazolam, and sevoflurane were discontinued after skin suturing. Sugammadex was administered intravenously at 2 mg/kg to reverse neuromuscular blockade in both groups; 0.2 mg flumazenil was administered intravenously as a reversal agent for remimazolam in the R group. By POD 2, IV patient-controlled analgesia was induced in both groups by administrating fentanyl (15 µg/kg) without regional nerve block.

### 2.5. Sample Size Calculation

In a previous study conducted on patients scheduled for total knee arthroplasty, the systolic BP was 144.9 ± 27.2 mmHg and 122.8 ± 22.6 mmHg in the remimazolam and sevoflurane groups, respectively, after induction of general anesthesia [21]. Based on these data, using G-power analysis (University of Dusseldorf, Dusseldorf, Germany), a sample size of 35 patients in each group was calculated with an α error of 0.05 and a power of 0.95. Considering a 10% dropout rate, the calculated target number of participants was 39 in each group for a total of 78 participants.

### 2.6. Statistical Analysis

Data were expressed as median (interquartile range) or number (%), as appropriate. The normality of continuous variables was evaluated using the Shapiro–Wilk test. Independent variable t-tests and Wilcoxon rank-sum tests were performed to analyze continuous variables with normal and non-normal distributions, respectively. The chi-squared test or Fisher’s exact test was used to analyze categorical variables. Univariate logistic regression analysis was used to identify the factors associated with hypotension during position change from supine to BCP, and all variables with *p* < 0.2 or previously described clinically important factors were included in the multivariate logistic regression analysis. Receiver operating characteristic (ROC) curve analysis was performed to determine the optimal cutoff value of baseline BP according to the occurrence of hypotension after transitioning to BCP using the maximum Youden index (sensitivity (%) + specificity (%) − 100). Statistical analyses were performed using SPSS software (version 22.0; IBM Corp., Armonk, NY, USA). Differences were considered statistically significant at two-tailed *p*-values < 0.05.

## 3. Results

### 3.1. Study Participants and Patient Characteristics

**Figure 1:**
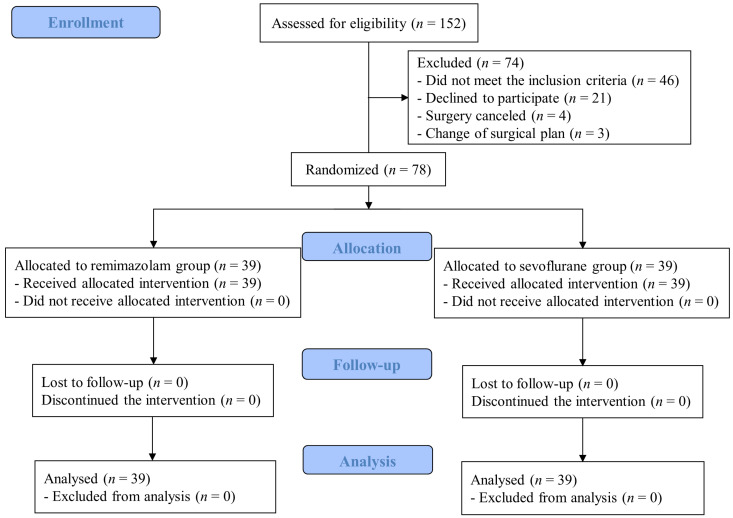
Study patient enrollment.

We recruited 152 patients, of whom 74 were excluded and 78 were randomly allocated to two groups of the same size. Thirty-nine patients in each group were analyzed without any loss to follow-up (Figure 1). No significant differences were observed between the study groups in terms of demographic data, medical history, preoperative laboratory test results, or surgical type (Table 1). No serious complications related to surgery or anesthesia occurred during the study period.

### 3.2. Primary Outcomes

The incidence of hypotension immediately after transition to a BCP was significantly lower in the R group than in the S group (7/39 (17.9%) vs. 16/39 (41.0%), *p* = 0.047) (Table 2). In logistic regression analysis, the risk factors associated with hypotension were sevoflurane administration and high baseline systolic BP (Table 3, Figure S1). The variables included in the multivariate logistic regression analysis (*p* < 0.2) were female sex, sevoflurane use, baseline systolic BP, and baseline HR.

### 3.3. Secondary Outcomes

**Figure 2:**
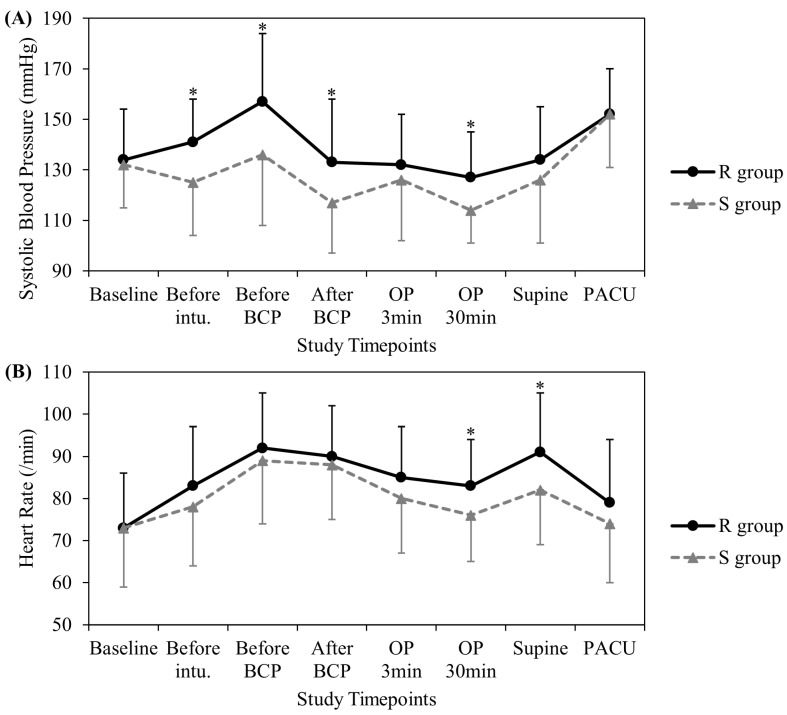
Trend in systolic blood pressure and heart rate at different study timepoints. (**A**) Systolic blood pressure. (**B**) Heart rate. Data are represented as means ± standard deviations. Statistically significant differences between the study groups at each timepoint are indicated as * *p* < 0.05. BCP, beach chair position; intu., intubation; OP, operation; PACU, post-anesthetic care unit.

The perioperative systolic BP and HR were significantly higher in the R group than in the S group at certain timepoints (Figure 2). The PVi was comparable between the two groups during the perioperative period (Figure S2; Table S1).

The intraoperative fluid volume, additional sedative administration, estimated blood loss, and surgical time were similar in both groups. The amount of intraoperative remifentanil and number of antihypertensive agents administered were significantly higher in the R group than in the S group. Vasopressors were administered more frequently to the S group than to the R group. The time from postoperative sugammadex administration to endotracheal tube extubation was significantly shorter in the R group than that in the S group. There were no significant differences in the time taken to recover to an Aldrete score of ≥9 in the PACU, postoperative AKI, PONV, PPC until POD 2, and postoperative hospital LOS between the two groups (Table 2).

**Figure 3:**
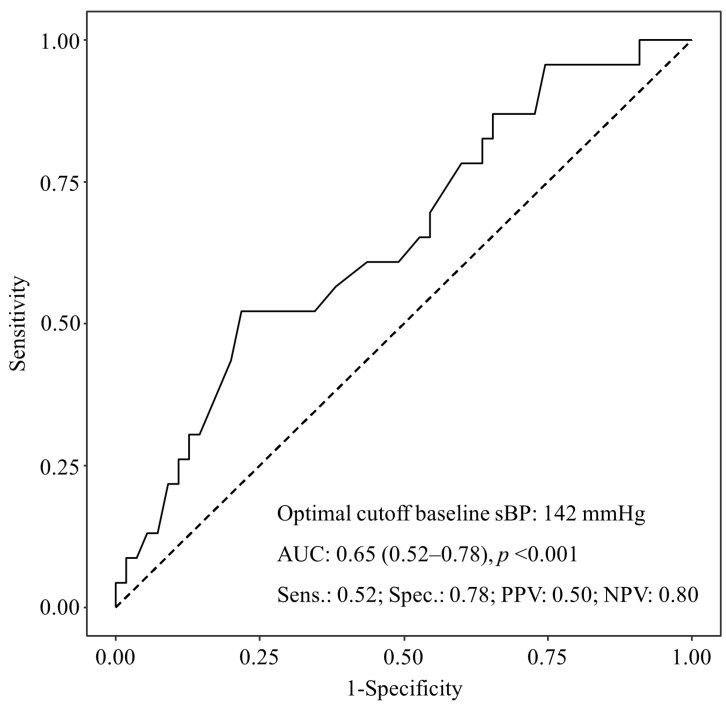
Receiver operating characteristic curve analysis of baseline systolic blood pressure relative to occurrence of hypotension after switching to the beach chair position. AUC, area under the curve; NPV, negative predictive value; PPV, positive predictive value; sBP, systolic blood pressure; Sens., sensitivity; Spec., specificity.

In the ROC curve analysis of baseline systolic BP relative to the occurrence of hypotension after the transition to a BCP, the cutoff value for systolic BP was 142 mmHg (Figure 3).

## 4. Discussion

This prospective, single-blind, randomized controlled trial evaluated the incidence of hypotension after transitioning to the BCP with remimazolam or sevoflurane as anesthetic agents in patients undergoing orthopedic surgery. When remimazolam was administered as an anesthesia induction and maintenance agent, hypotension occurred less frequently after switching to BCP. The perioperative BP and HR tended to remain higher in the remimazolam group than in the sevoflurane group at several timepoints. These results are consistent with those of several previous studies on the hemodynamics of remimazolam [21,22,23,24,25]. A feature of this study is that these hemodynamic stabilities were effective even during the transition to the BCP.

One notable outcome of this study was that participants with a high baseline BP had a higher incidence of hypotension after switching to a BCP. Previous studies reported a history of hypertension or high baseline BP as risk factors for the development of intraoperative hypotension [26,27,28]. In this study, patients taking antihypertensive medications were excluded to evaluate perioperative BP as the main outcome. Hypotension is prevalent in patients with a high baseline BP. This may be because some patients were not yet diagnosed with hypertension. Alternatively, the patient’s anxiety, which may have been reflected in the preoperative BP, could have temporarily increased baseline BP. Upon the induction of general anesthesia, anxiety was relieved, resulting in a >20% decrease in BP after switching to the BCP, thus meeting the criteria for hypotension in this study [29,30]. Additionally, through ROC curve analysis, we could derive a cutoff value for high baseline BP. Although the area under the curve was relatively low, it was significant for suggesting a clinically important cutoff value. Further research should be conducted on patients’ preoperative anxiety and BP fluctuations after switching surgical positions.

Previous studies have reported risk factors associated with hypotension that occur after switching to BCP, including old age, high BMI, history of hyperlipidemia, and hypertension [31,32,33]. Additionally, risk factors for intraoperative hypotension include female sex, administration of propofol as an anesthesia induction agent, high ASA-PS class, and high baseline HR and SVV [26,27,34,35,36,37]. However, in this study, age, sex, ASA-PS class, BMI, hyperlipidemia, and preoperative HR were not significantly associated with the occurrence of hypotension after switching to the BCP. Since extreme values such as ASA-PS class ≥ IV and BMI > 35 kg/m^2^ were excluded in this study and the sample size was relatively small, significant results may not have been achieved. SVV could not be measured because additional arterial cannulation could not be performed exclusively for this study, and PVi was measured noninvasively; however, there was no significant association with the occurrence of hypotension.

There was no significant difference in PVi between the two groups. First, remimazolam may not have a significant effect on fluid responsiveness compared to sevoflurane. A previous study also reported that there was no difference in PVi and SVV in the remimazolam group compared with other anesthetic drugs [38]. Systemic vascular resistance, which can affect fluid responsiveness, was also reported to be comparable [15]. Second, this may be a limitation of non-invasive monitoring [39,40]. If PPV and SVV were monitored, a clear conclusion could be drawn. A limitation of this study is that further analysis of fluid responsiveness indices could not be conducted by measuring only the PVi; therefore, further research measuring PPV and SVV together should be conducted.

Postoperative extubation time was shorter in the R group than in the S group. Remimazolam is a reversal agent, flumazenil [13]. Previous studies on remimazolam have reported that when flumazenil is administered postoperatively, the emergence time is shorter than that with traditional anesthetic drugs [41,42,43]. In the absence of flumazenil, the emergence time was not different or longer [44,45]. The results of the present study can also be attributed to the administration of flumazenil during the emergence phase. The presence of a reversal agent is a major advantage of remimazolam, and unless flumazenil is contraindicated, such as in seizures or arrhythmias, its administration is recommended during the emergence phase [46]. In this study, flumazenil was administered to the R group, and all 39 patients recovered from general anesthesia without serious complications. By shortening the emergence time through the administration of flumazenil, the economical and efficient distribution of medical facilities can be achieved.

Postoperative complications and hospital LOS were comparable between the two groups. We confirmed that remimazolam can be safely administered, similar to sevoflurane. In a previous study conducted by the authors [21], the incidence of AKI and PPC did not differ between the remimazolam and sevoflurane groups. Similar results were obtained in the present study, and the incidences of AKI and PPC were quite low because the study was conducted in relatively young and healthy patients. The authors expected that the incidence of PONV would be lower with IV anesthetic remimazolam than with inhaled anesthetic sevoflurane [47], but there was no difference between the two groups. Wei et al. reported that flumazenil administration increased the incidence of PONV [48]. In this study, flumazenil was administered to the remimazolam group, which would have increased the incidence of PONV; therefore, the incidence of PONV was comparable between the groups. Further studies should be conducted on remimazolam, flumazenil, and PONV incidence. In clinical practice, the risks and benefits of flumazenil should be considered to shorten emergence time and reduce PONV.

This study has several limitations. First, the sample size was relatively small and double-blinding could not be performed owing to significant differences in the administration methods of remimazolam and sevoflurane. However, assessor blinding reduced the bias caused by group allocation during postoperative data evaluation. Furthermore, although this was a small study, significant differences between the two groups were observed. In addition, determining the exact sample size was difficult. Few previous studies have compared the incidence of hypotension after the administration of remimazolam after switching surgical position. Alternatively, the sample size was calculated based on the systolic BP used to define hypotension. The power calculated from the provided outcomes is 0.61, which causes a considerably high probability of a type II error. Although type II errors may be relatively high, there was a significant difference between groups, and meaningful outcomes were obtained. This study will serve as a reference for further research on the occurrence of hypotension associated with remimazolam. Second, the types of surgeries used were not uniform. However, regardless of the type of surgery, hypotension was assessed immediately after posture switching to the BCP, and there was no significant difference in the type of surgery between the two groups. In addition, a single surgeon performed all surgeries during the study, and several types of surgeries were included to generalize the results of the transition to the BCP. Third, arterial BP, SVV, and PPV were not monitored using arterial cannulation in any participant. No additional invasive procedures were performed on the participants in this study. Non-invasive PVi monitoring was performed instead of SVV and PPV for hemodynamic monitoring. Lastly, a type I error may have occurred owing to multiple comparisons of BP at various timepoints during the study. Measuring and analyzing overall BP is difficult. Rather than accepting the results of this study as an overall difference in BP, these results should be used as a reference for managing BP at various timepoints during surgery.

## 5. Conclusions

In conclusion, remimazolam compared with sevoflurane, as a maintenance agent for general anesthesia, can reduce the occurrence of hypotension when switching to the BCP. In addition, hypotension frequently occurs after switching to BCP in patients with high baseline systolic BP. Therefore, for patients with high baseline BP or those expected to develop hypotension after changing to BCP, remimazolam should be considered as a general anesthesia induction and maintenance agent. Further large-scale, double-blind, randomized controlled trials should be conducted in patients undergoing surgery in the BCP with remimazolam.

## Figures and Tables

**Table 1:** Demographic data of the study cohort.

	R Group (*n* = 39)	S Group (*n* = 39)	*p*-Value
Age (y)	60 (48, 69)	60 (45, 70)	0.984
Sex (male/female) (*n*)	25 (64.1%)/ 14 (35.9%)	24 (61.5%)/ 15 (38.5%)	1.000
BMI (kg/m^2^)	25.0 ± 3.1	25.1 ± 2.8	0.792
ASA-PS class (I/II/III)	10 (25.6%)/ 26 (66.7%)/ 3 (7.7%)	9 (23.1%)/ 28 (71.8%)/ 2 (5.1%)	0.849
Diabetes mellitus (*n*)	6 (15.4%)	9 (23.1%)	0.566
Dyslipidemia (*n*)	13 (33.3%)	13 (33.3%)	1.000
Smoking (non/ex/current) (*n*)	28 (71.8%)/ 5 (12.8%)/ 6 (15.4%)	26 (68.4%)/ 3 (7.9%)/ 9 (23.7%)	0.560
White blood cell (×10^3^/μL)	6.08 (5.18, 7.38)	6.75 (5.56, 8.19)	0.261
Hematocrit (%)	41.5 ± 3.3	41.6 ± 3.3	0.905
Platelet (×10^3^/μL)	228 ± 54	223 ± 44	0.682
INR	0.95 ± 0.06	0.95 ± 0.06	0.750
Bilirubin (mg/dL)	0.66 (0.47, 0.81)	0.66 (0.52, 0.80)	0.738
Creatinine (mg/dL)	0.82 (0.67, 0.91)	0.80 (0.67, 0.89)	0.693
Type of surgery (*n*)			0.971
Shoulder arthroscopy	20 (51.28%)	18 (46.15%)	
Fixative removal	6 (15.38%)	7 (17.95%)	
ORIF	5 (12.82%)	5 (12.82%)	
Total shoulder arthroplasty	8 (20.51%)	9 (23.08%)	

Normally distributed variables are presented as mean ± standard deviation (SD) and non-normally distributed as median with interquartile range (IQR) or number (%). ASA-PS, American Society of Anesthesiologists physical status; BMI, body mass index; INR, international normalized ratio; ORIF, open reduction and internal fixation; R, remimazolam; S, sevoflurane.

**Table 2:** Perioperative outcomes.

	R Group (*n* = 39)	S Group (*n* = 39)	*p*-Value
Intraoperative data			
Dose of remimazolam			
Induction (mg/kg)	0.12 (0.10, 0.14)	N/A	
Maintenance (mg/kg/h)	1.11 (0.93, 1.21)	N/A	
Dose of propofol (mg/kg)	N/A	1.60 (1.48, 1.75)	
Mean sevoflurane (vol%)	N/A	2.10 (2.00, 2.25)	
Remifentanil infusion (µg/kg/min)	0.10 (0.06, 0.14)	0.08 (0.05. 0.10)	0.035 *
Vasopressor administration (*n*)	10 (25.6%)	22 (56.4%)	0.011 *
Phenylephrine (µg/kg) ^†^	2.0 (1.3, 3.8)	4.2 (1.6, 9.7)	0.092
Ephedrine (mg) ^†^	0 (0, 0)	0 (0, 10)	0.172
Antihypertensive administration (*n*)	11 (28.2%)	3 (7.7%)	0.039 *
Nicardipine (mg) ^‡^	2.0 (0.9, 2.4)	0.5 (0.4, 0.8)	0.117
Esmolol (mg/kg) ^‡^	0 (0, 0)	0 (0, 0)	0.523
Sedative administration (*n*)	2 (5.1%)	0	0.474
Hypotension after BCP (*n*)	7 (17.9%)	16 (41.0%)	0.047 *
Fluid administration (mL/kg/h)	2.4 (1.9, 3.1)	2.1 (1.6, 2.8)	0.202
Estimated blood loss (mL)	50 (30, 70)	30 (30, 50)	0.329
Surgical time (min)	65 (45, 80)	65 (40, 85)	0.984
Postoperative data			
Extubation time (s) ^¶^	210 (159, 264)	322 (198, 458)	0.001 *
Aldrete score ≥ 9 at PACU (min)	30 (30, 39)	30 (30, 35)	0.892
Acute kidney injury (*n*)	1 (2.6%)	0	1.000
PONV (*n*)	7 (17.9%)	7 (17.9%)	1.000
PPC (*n*)	0	1 (2.6%)	1.000
Hospital length of stay (d)	2 (2, 3)	2 (2, 4)	0.294

Data are presented as medians (interquartile ranges) or numbers (%). * Statistically significant. ^†^ Ephedrine and phenylephrine were analyzed in patients administered vasopressors. ^‡^ Nicardipine and esmolol were analyzed among the patients administered antihypertensive agents. ^¶^ Extubation time refers to the time from the administration of sugammadex to extubation of the endotracheal tube after surgery. BCP, beach chair position; N/A, not applicable; PACU, post-anesthetic care unit; PONV, postoperative nausea and vomiting; PPC, postoperative pulmonary complications; R, remimazolam; S, sevoflurane.

**Table 3:** Univariate and multivariate logistic regression analyses of factors associated with hypotension after transitioning to the beach chair position.

	Univariable		Multivariable	
	OR [95% CI]	*p*-Value	OR [95% CI]	*p*-Value
Age	1.02 [0.98–1.05]	0.349	1.02 [0.97–1.07]	0.435
Female	2.44 [0.90–6.73]	0.080	1.55 [0.42–5.75]	0.505
ASA-PS				
I	Reference			
II	0.66 [0.22–2.06]	0.460		
III	0.43 [0.02–3.68]	0.486		
Body mass index	1.02 [0.86–1.21]	0.820	1.03 [0.83–1.28]	0.811
Diabetes mellitus	1.25 [0.35–4.06]	0.717		
Dyslipidemia	1.10 [0.38–3.02]	0.861	0.73 [0.19–2.57]	0.636
Sevoflurane group	3.18 [1.16–9.45]	0.029 *	3.88 [1.28–13.32]	0.021 *
Baseline systolic BP	1.03 [1.00–1.07]	0.027 *	1.04 [1.00–1.07]	0.037 *
Baseline heart rate	1.03 [0.99–1.07]	0.189	1.02 [0.98–1.07]	0.405
Baseline PVi	0.96 [0.88–1.06]	0.429		

* Statistically significant. ASA-PS, American Society of Anesthesiologists physical status; BP, blood pressure; CI, confidence interval; OR, odds ratio; PVi, pleth variability index.

## Data Availability

The datasets used and analyzed during the current study are available from the corresponding author upon reasonable request. The data are not publicly available because of privacy or ethical restrictions.

## Supplementary Materials

The following supporting information can be downloaded at: https://www.mdpi.com/article/10.3390/jcm13082364/s1, Figure S1: Forest plot of the odds ratios for occurrence of hypotension after switching to the beach chair position; Figure S2: Trend in Pleth variability index at different study timepoints; Table S1: Perioperative blood pressure, heart rate, and Pleth variability index.
