# Supplementary material for: Comparison of Hemodynamic Parameters Based on the Administration of Remimazolam or Sevoflurane in Patients under General Anesthesia in the Beach Chair Position: A Single-Blinded Randomized Controlled Trial
Source: J Clin Med. 2024 Apr 18;13(8):2364. doi: 10.3390/jcm13082364 (PMC11051199; doi:10.3390/jcm13082364)
Supplement: Supplementary file 1 [file jcm-13-02364-s001.zip › jcm-2955889-supplementary.pdf]

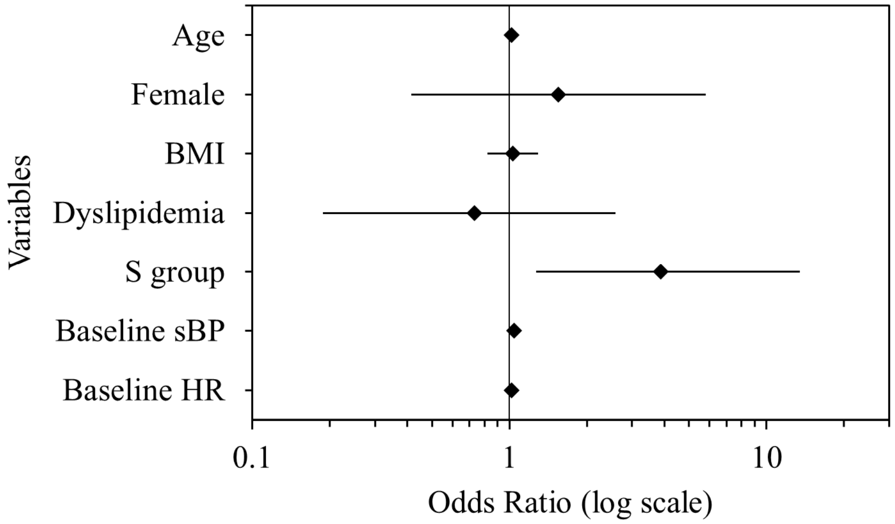

Figure S1. Forest plot of the odds ratios for occurrence of hypotension after switching to the beach chair position.

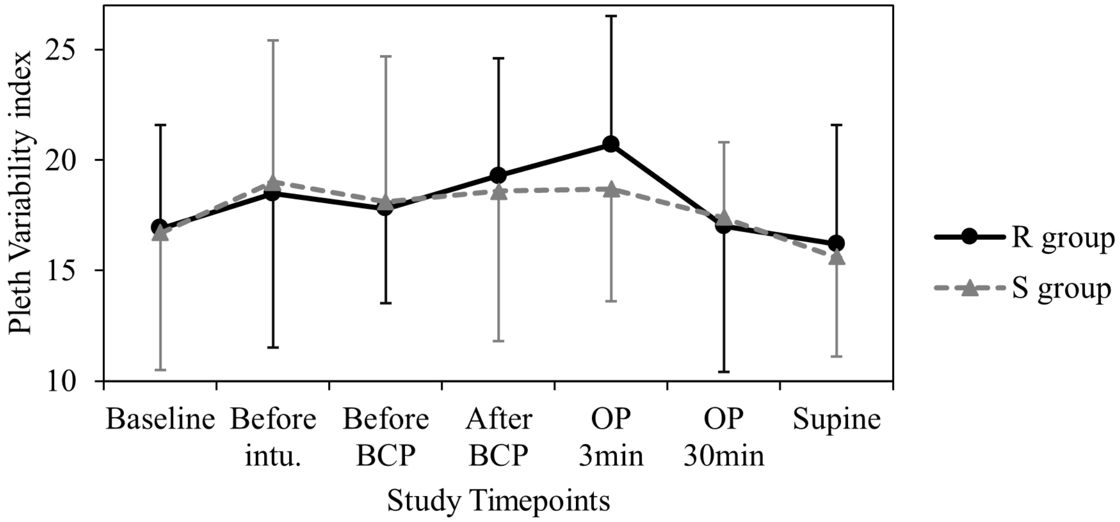

Figure S2. Trend in Pleth variability index at different study timepoints.

Table S1. Perioperative blood pressure, heart rate, and pleth variability index.

	R group (*n* = 39)	S group (*n* = 39)	*p*-Value
Systolic blood pressure (mmHg)			
Baseline	134 ± 20	132 ± 17	0.599
Before tracheal intubation	141 ± 17	125 ± 21	<0.001*
Before the BCP	157 ± 27	136 ± 28	0.001*
After switching to the BCP	133 ± 25	117 ± 20	0.002*

Surgical incision	132 ± 20	126 ± 24	0.195
30 min after incision	127 ± 18	114 ± 13	<0.001*
After supine	134 ± 21	126 ± 25	0.127
Post-anesthetic care unit	152 ± 18	152 ± 21	0.935
Mean blood pressure (mmHg)			
Baseline	100 ± 14	99 ± 12	0.739
Before tracheal intubation	104 ± 12	94 ± 16	0.002*
Before the BCP	114 ± 21	101 ± 17	0.004*
After switching to the BCP	100 ± 18	90 ± 15	0.007*
Surgical incision	101 ± 16	99 ± 21	0.579
30 min after incision	95 ± 14	87 ± 11	0.009*
After supine	98 ± 18	91 ± 21	0.090
Post-anesthetic care unit	108 ± 18	106 ± 16	0.589
Diastolic blood pressure (mmHg)			
Baseline	83 ± 15	83 ± 11	0.870
Before tracheal intubation	83 ± 11	74 ± 15	0.005*
Before the BCP	89 ± 20	78 ± 17	0.009*
After switching to the BCP	82 ± 16	72 ± 14	0.003*
Surgical incision	84 ± 15	81 ± 21	0.484
30 min after incision	75 ± 13	70 ± 12	0.084
After supine	75 ± 14	69 ± 20	0.138
Post-anesthetic care unit	85 ± 16	83 ± 16	0.559
Heart rate (/min)			
Baseline	73 ± 13	73 ± 14	0.953
Before tracheal intubation	83 ± 14	78 ± 14	0.115
Before the BCP	92 ± 13	89 ± 15	0.299
After switching to the BCP	90 ± 12	88 ± 13	0.549
Surgical incision	85 ± 12	80 ± 13	0.101
30 min after incision	83 ± 11	76 ± 11	0.005*
After supine	91 ± 14	82 ± 13	0.008*
Post-anesthetic care unit	79 ± 15	74 ± 14	0.091
Pleth variability index			
Baseline	16.9 ± 4.7	16.7 ± 6.2	0.869
Before tracheal intubation	18.5 ± 7.0	19.0 ± 6.4	0.776
Before the BCP	17.8 ± 4.3	18.1 ± 6.6	0.872
After switching to the BCP	19.3 ± 5.3	18.6 ± 6.8	0.618

Surgical incision	20.7 ± 5.8	18.7 ± 5.1	0.110
30 min after incision	17.0 ± 6.6	17.4 ± 3.4	0.698
After supine	16.2 ± 5.4	15.6 ± 4.5	0.637

Data are presented as means ± standard deviations. *Statistically significant. BCP, beach chair position; R, remimazolam; S, sevoflurane.
